# In Vivo Measurement of Wrist Movements during the Dart-Throwing Motion Using Inertial Measurement Units

**DOI:** 10.3390/s21165623

**Published:** 2021-08-20

**Authors:** Gabriella Fischer, Michael Alexander Wirth, Simone Balocco, Maurizio Calcagni

**Affiliations:** 1 Division of Plastic Surgery and Hand Surgery, University Hospital Zurich, University of Zurich, Raemistrasse 100, 8091 Zurich, Switzerland; michael.a.wirth@bluewin.ch (M.A.W.); maurizio.calcagni@usz.ch (M.C.); 2 Institute for Biomechanics, ETH Zurich, Leopold-Ruzicka-Weg 4, 8093 Zurich, Switzerland; 3 Department of Mathematics and Informatics, University of Barcelona, Gran Via 585, 08007 Barcelona, Spain; simone.balocco@ub.edu; 4 Computer Vision Center, 08193 Bellaterra, Spain

**Keywords:** inertial measurement units, kinematics, motion analysis, optoelectronic motion capture, wrist, range of motion, dart throwing motion, activities of daily living

## Abstract

Background: This study investigates the dart-throwing motion (DTM) by comparing an inertial measurement unit-based system previously validated for basic motion tasks with an optoelectronic motion capture system. The DTM is interesting as wrist movement during many activities of daily living occur in this movement plane, but the complex movement is difficult to assess clinically. Methods: Ten healthy subjects were recorded while performing the DTM with their right wrist using inertial sensors and skin markers. Maximum range of motion obtained by the different systems and the mean absolute difference were calculated. Results: In the flexion–extension plane, both systems calculated a range of motion of 100° with mean absolute differences of 8°, while in the radial–ulnar deviation plane, a mean absolute difference of 17° and range of motion values of 48° for the optoelectronic system and 59° for the inertial measurement units were found. Conclusions: This study shows the challenge of comparing results of different kinematic motion capture systems for complex movements while also highlighting inertial measurement units as promising for future clinical application in dynamic and coupled wrist movements. Possible sources of error and solutions are discussed.

## 1. Introduction

Kinematic motion analysis is widely used in clinical settings and research to determine the range of motion (ROM) of joints with the purpose of quantifying the degree of impairment of the joint, planning rehabilitation strategies, and assessing the effect of operative interventions [1]. The standard movement planes of flexion–extension and radial–ulnar deviation are usually assessed for the wrist joint. However, movements in the so-called “dart-throwing” plane of motion (DTM) have gained clinical interest as they are carried out during several functional activities of daily living [2,3,4]. For example, hammering a nail, twisting the lid of a jar and pouring from a jug all cause wrist movements in the DTM plane [5]. The wrist moves in an arch from radial-extension to ulnar-flexion during the DTM with most motion anatomically occurring in the midcarpal joint and the proximal carpal row remaining relatively immobile, which for example affects clinical decision making between different techniques of partial wrist fusion [2]. Wrist movements in this plane are unique to human wrists and have provided a significant evolutionary advantage [5,6].

Due to the complexity of this combined movement, different kinematic motion analysis techniques have been used to measure it, ranging from imaging techniques such as computed tomography and magnetic resonance imaging to marker-based optoelectronic motion capture systems [3,4,5]. The latter currently represents the most common reference standard for motion analysis [1]. While imaging techniques have limitations due to radiation and restrictions in image acquisition speed [3], optoelectronic motion capture systems (OMC) are still expensive and only feasible in a specialized laboratory [1]. The accuracy of the position determination is very high (<0.1 mm) [7], but the choice of the marker set and kinematic models influences the calculated joint angles mainly due to soft tissue artifacts. There is currently no standardized method for wrist measurement.

Inertial measurement units (IMU) do not require a specialized laboratory and are much cheaper in comparison. They are based on either a combination of accelerometers, gyroscopes, and magnetometers; a combination of two of them; or one of these sensors alone. Used in combination, the different sensors can compensate for the limitations of each other and provide reliable measurements [8]. The accuracy of IMU is dependent on the exact sensor and software specification used. In a previous study, we investigated an IMU system for measurement of basic motion tasks of the wrist and found it to be feasible for clinical application with similar measurements to the gold standard, an OMC based system [9]. However, as task-specific validation of a new system is necessary for possible clinical application, the high accuracy in basic motion tasks cannot be automatically assumed for more complex movements of daily living such as the DTM [1].

ROM measurements in a clinical setting are usually performed using a goniometer to measure maximal joint mobility in the anatomical planes, but clinicians are interested in reliable measurements of more complex movements such as the DTM, as they might be a better predictor of wrist function in daily living. Approaches for measuring the DTM ROM using a goniometer have been described but are not performed in everyday clinical practice [10]. The DTM is not measured in current clinical practice and during hand therapeutic treatment sessions, but with the application of IMU to patients during their treatment sessions, wrist movements in the practiced activities of daily living such as the DTM could be assessed and monitored in real-time without further effort. Furthermore, there have also been attempts to calculate the DTM ROM using the ROM in the anatomical planes, but this approach has only been tested in healthy subjects [11]. Reissner et al. have shown that the ROM of the DTM in patients after partial wrist fusion does not correlate with the ROM in the anatomical planes [2]. Therefore, direct measurement of DTM in patients is necessary to provide accurate information about the mobility of the wrist in the functionally important DTM plane.

IMU based measurements pose a possible solution for direct measurement of complex three-dimensional tasks [12]. They are easy enough for a clinical use without the need for an extensive laboratory like the OMC based solutions and do not expose patients to radiation like imaging techniques [12]. An open question is how IMU cope with the high movement speeds occurring in the DTM. Previous studies have shown that the accuracy of IMU is dependent on the movement speed, with higher speeds being associated with higher errors [13,14]. However, task-specific analysis of the DTM in the wrist is missing.

The aim of this study is to compare an IMU already validated for basic motion tasks for the more complex combined wrist movements occurring during the DTM with the current gold standard of motion analysis, an OMC. The IMU DyCare^®^ Lynx, developed for clinical use, was selected and compared to an OMC system by Vicon^®^.

## 2. Materials and Methods

The current analysis of the DTM is part of a larger motion analysis project. The DTM, described in this paper, and basic motion task measurements presented in a previous publication [9], were recorded in the same measurement sessions. The common aspects of the methodology are only briefly summarized here. For a more detailed description, we refer to [9]. The dart-throwing motion analyzed in this paper (Section 2.3 Experimental Protocol) and the project-specific data analysis (Section 2.6 Data Analysis) are described in detail below.

### 2.1. Participants

Ten healthy subjects (5 female, 5 male, 24.4 ± 2.2 years) with right dominant hands were included in the study. Informed consent was given by all test persons for their data to be used for research purposes and all their related data were anonymized. This study was conducted in accordance with the Declaration of Helsinki and was carried out with permission from the local ethics committee of the Canton of Zurich, Switzerland (Kek-ZH-Nr: 2018-00457). For more details about participants and inclusion/ exclusion criteria we refer to [9]. Each subject’s measurement was repeated on a different day.

### 2.2. Setup

**Figure 1:**
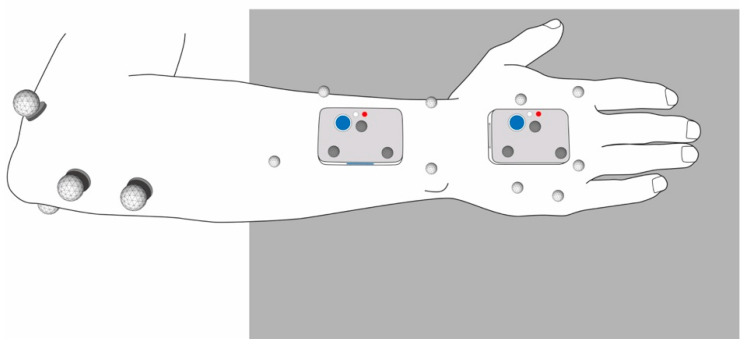
Sensor (IMU), skin marker (light grey, OMC1), and sensor marker (dark grey, OMC2) placement.

Participants’ right arm and hand were equipped with the two IMU and the OMC skin markers as described in [9] (Figure 1). The OMC skin markers were applied to anatomical landmarks on the hand and forearm of the test persons in the same configuration we used in a previous study [9], based on an approach for which we have verified the repeatability in a test-retest study [15].

Two different OMC marker setups were compared in this study. For the first one referred to as OMC1, participants were equipped with four reflective markers of 9 mm diameter on the elbow, four 5 mm markers on the forearm and five 5 mm markers on the dorsal aspect of the hand. These markers are shown in light grey in Figure 1. For the second setup referred to as OMC2, three 5 mm markers were attached directly to each of the two IMU sensors, as shown in dark grey in Figure 1.

For IMU measurements, a well-defined and repeatable configuration process is crucial. The longitudinal axis of the sensor on the hand was aligned along the metacarpal bone of the middle finger. The sensor on the forearm was placed slightly proximal to the wrist and aligned along the longitudinal axis of the forearm. A detailed description of the configuration and the applied criteria can be found in our previous study [9].

### 2.3. Experimental Protocol

After attaching the markers and sensors, a static reference position and a set of basic motion tasks [9] as well as a set of five trials of the dart throwing motion (DTM) were recorded simultaneously with the IMU and OMC. The measurements of both systems were started and stopped at approximately the same time by the examinators. However, no exact synchronization of the measurements was performed at this point.

**Figure 2:**
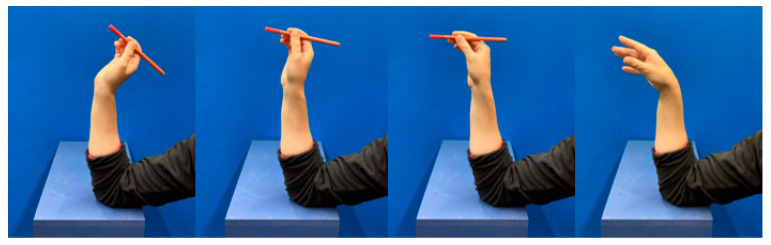
Sequence of the dart-throwing motion task.

For the DTM trials, test subjects were seated in front of the examination table (105 cm high) with their right forearm placed in front of them on the examination table, the wrist being in a neutral position. They were then instructed to grab a small cylindrical object also lying on the table, prop up their arm on the elbow on the table, throw the object from their wrist as they would naturally throw a dart without lifting the elbow from the table, and then place the arm in the starting position. During the DTM, the elbow of the participants was sitting on the table in front of them and they had their forearm in an upright position, throwing the object from their wrist (see Figure 2). As a result, the distance of the hand to the table varied depending on the length of the forearm. A custom-made wooden table was used to reduce possible interference from metal parts on the magnetometer.

### 2.4. Technologies Used

The OMC (VICON^®^ MX3+ and VICON^®^ MX3 motion capture system, Oxford Metrics Ltd., Oxfordshire, UK) used eleven cameras with a resolution of 659 × 493 pixel, recording at a frequency of 100 Hz. Data were collected and processed in the software provided by the manufacturer VICON^®^ Nexus (version 2.3).

The IMU (DyCare^®^ Lynx, Manufacturer: DyCare, Barcelona, Spain) used two identical sensors with dimensions of 50 mm × 34 mm × 14 mm, each consisting of a gyroscope, accelerometer, and magnetometer and recording at a sampling frequency of 102.4 Hz. They were configured in the following way: gyroscope: 2000°/s, accelerometer: 2 g, magnetometer 4.7 Ga. The sensors were calibrated according to the manufacturer’s protocol before each measurement and set to the “free joint” setting. The calibration was carried out on the measuring table where the measurements would later be performed. Data of the IMU system were collected and processed in the software DyCare^®^ Lynx (version 1.7.0) provided by the manufacturer.

### 2.5. Kinematic Analysis

#### 2.5.1. OMC Data

The recorded data of the OMC were processed in VICON^®^ Nexus, where all markers were manually labelled. Small gaps in marker recordings were filled using VICON^®^ Nexus’ built-in gap filling routine. Further data analysis was carried out in Matlab (R2016b) using the same approach described in [9], which is based on marker clusters considered as rigid bodies with the wrist joint centers determined by a functional approach [15,16,17], with the joint center simulated as a ball joint and the flexion axis as a hinge joint. Joint movements were calculated according to [18]. This analysis was performed for the marker clusters on the skin (OMC1) as well as the markers on the IMU sensors (OMC2).

#### 2.5.2. IMU Data

The kinematic evaluation of the IMU was performed by the included software of the DyCare^®^ device. In the kinematic approach of the IMU, the axes of the wrist are determined by the main axes of the proximal sensor (geometric approach) and the zero angle of the wrist is determined by an offset in the neutral reference position. The wrist angles were imported into Matlab (R2016b) for further comparison. Wrist joint flexion and radial deviation are presented as positive angles whereas extension and ulnar deviation are presented as negative angles in all trials of all measurement methods.

### 2.6. Data Analysis

The angle curves derived from both OMC and IMU were then processed in the same way. An offset correction was performed, assuming that the reference position defines the “zero position” in the wrist. Consequently, the measured wrist angle at the reference position was subtracted from the whole curve of each dynamic trial of each measurement system. Slightly different sampling rates (104 Hz IMU, 100 Hz OMC, both equally distributed) were adapted by data interpolation of the IMU signal.

Since the two measuring systems were not synchronized in time during data acquisition, a temporal alignment of the signals was subsequently performed using a rigid translation. First, the signal was reduced to the actual throwing movement by cutting it to 500 ms before and after the maximum derivative of the flexion angle (duration of 1000 ms in total). Then, the optimal alignment of the cropped derivative signals derived from OMC1 and IMU was calculated using the built-in cross correlation function xcorr in Matlab. The maximum location of the cross-correlation measure to align was determined. The IMU signals were then shifted by the value determined by cross correlation and cut again to the point of interest of 1000 ms. The resulting wrist flexion–extension and radial–ulnar deviation angle curves of all three measurement systems, now cut and aligned, were used for further comparison.

The derivative of the angle curves was calculated, and its signal noise was reduced using a Gaussian filter (sigma value = 10). The root-mean-squared error (RMSE) of the wrist angles and the derivatives between different acquisition systems was calculated as follows:

(1) $${\mathrm{RMSE}}_{jk}=\sqrt{\sum_{i=1}^{n}\frac{{\left(\alpha_{jk}^{a}-\alpha_{jk}^{b}\right)}^{2}}{n}},$$

with *j* being the subject number, *k* the trial number, and *a*, *b* the acquisition systems. The range of motion (ROM) of each trial was calculated as follows:

(2) $${\mathrm{ROM}}_{jk}=M_{jk}-m_{jk},$$

with *j* being the subject number, *k* the trial number, M the maxima and m the minima identified.

The mean absolute difference (MAD) between ROM among the systems was calculated as follows:

(3) $${\mathrm{MAD}}_{jk}^{ab}=abs({\mathrm{ROM}}_{jk}^{a}-{\mathrm{ROM}}_{jk}^{b}),$$

with *j* being the subject number, *k* the trial number, and *a*, *b* the acquisition systems. Data of both measurement sessions were used to calculate above-described parameters.

**Figure 3:**
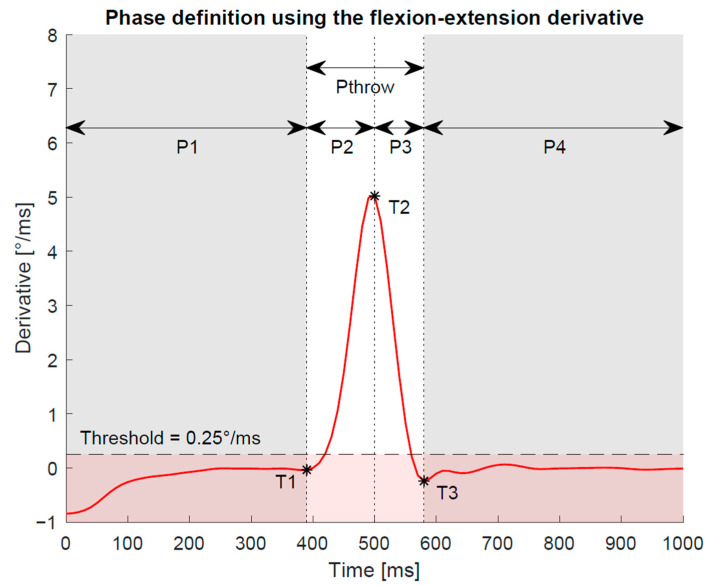
It was assumed that there is a relatively stable phase of the signal before (P1) and after (P4) the actual throw and that the throw movement is characterized by a high derivative. Hence, T1 was defined as the last time point before the peak derivative (T2), where the derivative lies below a certain threshold for more than 30 ms. Similarly, T3 was defined as the first time point after the peak derivative, where the derivative falls again below a certain threshold for more than 30 ms. Together, P2 and P3 represent the phase of the actual throw, P_throw_.

For analysis of the duration of the actual throwing movement, the start (T1) and end (T3) point were determined in each trial and for each measurement system based on the Gaussian filtered derivatives (Figure 3). The throwing phase was defined by the derivative exceeding a certain threshold for more than 30 ms before and after the maximum slope. The threshold value was set at 0.25°/ms for the flexion–extension movement. This corresponds to approximately 5% of the maximum derivative in this plane.

Similar to [5], the plane of the DTM was calculated by fitting a linear trend line of best fit to the plotted data of coupled wrist angles (flexion–extension against radial–ulnar deviation angle) for each DTM trial. Using standard trigonometric functions, the angle of the obtained regression line to the flexion axis was then calculated and compared between the three systems. As defined in [5], a threshold of the R2 value of 0.70 needs to be achieved for the trend line to be representative for global wrist movement. Furthermore, the percentage of maximum ROM (ROM_BMT_) (determined from the basic motion tasks [9]) against the ROM used during the DTM task (ROM_DTM_) was calculated for each system.

In addition, the relative motion of the OMC marker clusters was calculated for the hand and forearm segments. For this purpose, the position and orientation of the OMC1 palm and forearm segment were determined for each frame during the dynamic trials relative to the static reference position by means of a least-squares fit of the respective marker clusters [19]. The resulting rotation matrix and translation vector were then used to transform the measured marker positions into the coordinate system of the reference position. Subsequently, the relative movement of the centroids for the OMC1 and OMC2 marker clusters were determined with respect to the reference position for the hand and forearm segment. By means of a projection on the segmental coordinate axes [17], the relative movement of the cluster centroids was assigned to the anatomical planes. The MAD and the peak change in centroid distance was calculated. To detect possible task specific patterns, this relative movement of the marker clusters was analyzed for the dart throwing motion as well as for the basic motion tasks published earlier [9].

### 2.7. Statistical Analysis

From the total ROM_DTM_ values of the systems, MAD and coefficient of variation (CV) between the systems were calculated to assess the variability within the measurement as well as the measurement error for each system.

A Kruskal-Wallis test (α = 0.05) was used to assess the statistical significance of the calculated parameters among the three acquisition systems. Bonferroni correction for multiple comparisons was applied.

## 3. Results

**Figure 4:**
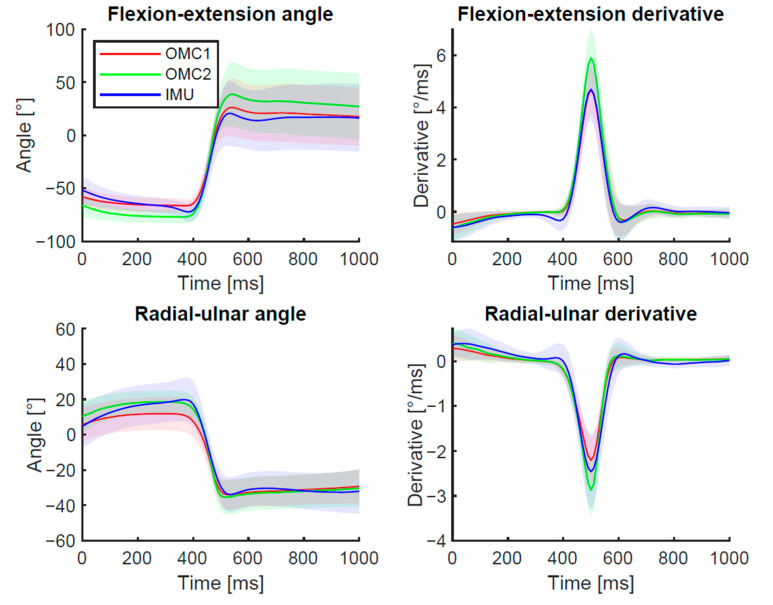
Mean (±SD) angles and derivatives over all trials and subjects for each measurement method. The signals are aligned using a rigid translation and cropped to 500 ms before and after the maximum derivative of the flexion angle. The derivative is filtered using a Gaussian filter.

**Figure A1:**
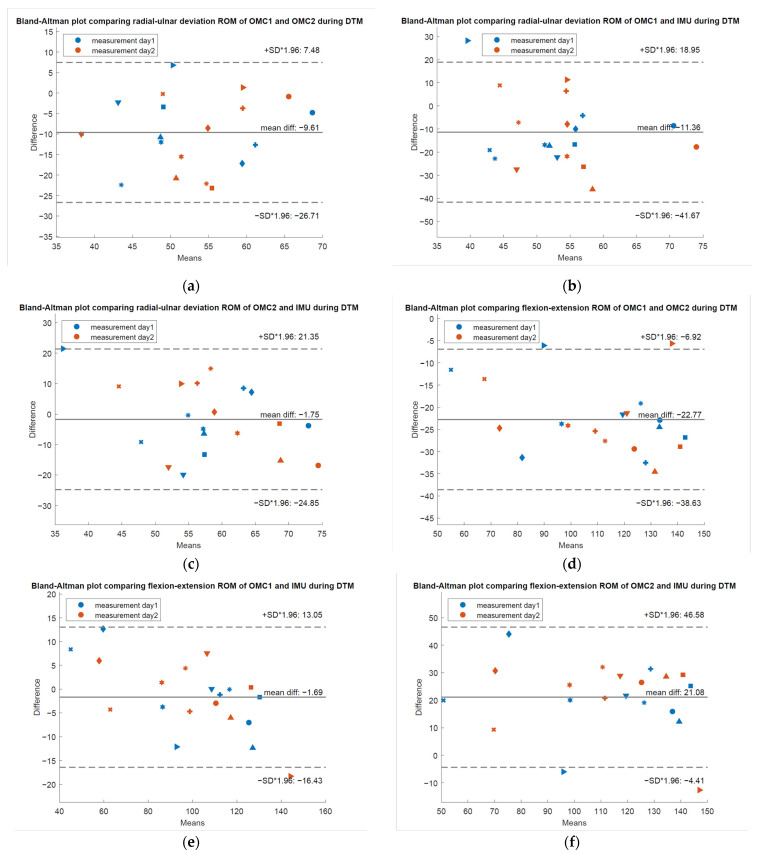
Bland-Altman plots showing the difference between two measurement methods for each individual trial (each trial is marked as a separate symbol) and for both measurement days in red and blue. (**a**) Comparison of OMC1 and OMC2 in radial–ulnar deviation, (**b**) comparison of OMC1 and IMU in radial–ulnar deviation, (**c**) comparison of OMC2 and IMU in radial–ulnar deviation, (**d**) comparison of OMC1 and OMC2 in flexion–extension, (**e**) comparison of OMC1 and IMU in flexion–extension, (**f**) comparison of OMC2 and IMU in flexion–extension.

The summarized results over all participants plotted for each measurement system are displayed in Figure 4. The results consider the aligned and cut signals only. An in-detail representation of the results of each individual test person is shown as Bland-Altman plots in Appendix A (Figure A1). For the OMC measurement, small marker gaps (<10 frames) occurred, which were filled by interpolation during post-processing with Vicon Nexus’ built-in function. However, in a single measurement session, marker gaps up to 40 frames were found during the fast phase of the DTM for the OMC1 captures. The IMU signal showed a continuous angle curve for all trials.

**Figure 5:**
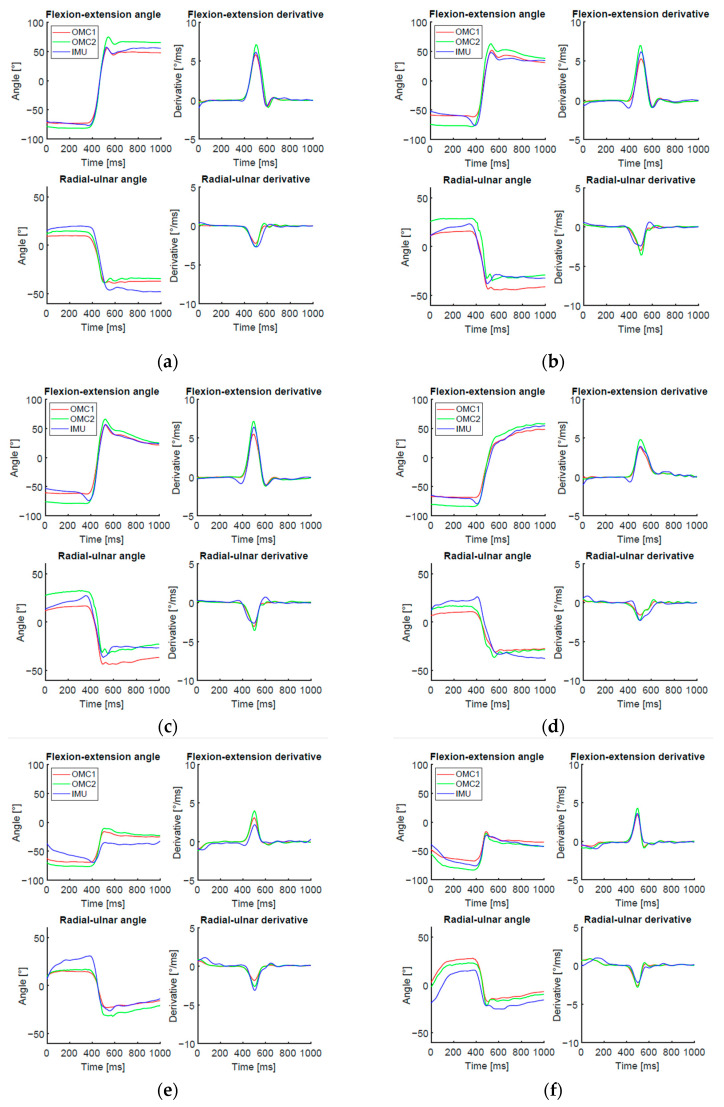
Examples of individual trials representing (**a**) standard case of the DTM, (**b**) additional wrist extension movement at the beginning of the throwing movement in the IMU signal approximately between 35-40ms while both OMC signals remain constant, (**c**) noise of the OMC2 signal, (**d**) flexion angle is maximal at the end of the selected signal, true maximal value is cut off, (**e**) difficulties in temporal alignment because of big differences in the shape of the signals, (**f**) small movement in the wrist without reaching positive joint angles (corresponding to no wrist flexion) while throwing the object.

Figure 5 shows an individual trial representing a standard case of DTM as well as examples of unexpected cases. Among the identified issues were the following: additional wrist extension movement at the beginning of the throw in the sensor signal (approximately between 35–40 ms), noise of the OMC2 signal, and location of the maximum flexion angle at the end of the selected signal. Additionally, some subjects performed only a small movement in the wrist without reaching positive flexion angles while throwing the dart.

**Figure 6:**
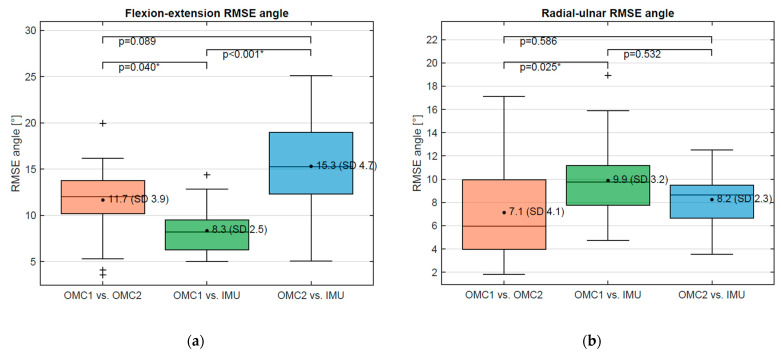
(**a**) RMSE of flexion–extension angle between OMC1, OMC2, and IMU acquisition systems and (**b**) RMSE of radial–ulnar deviation angle between OMC1, OMC2, and IMU acquisition systems. For Values marked as *, the difference is statistically significant. The symbols are statistical outliers and can be captioned as such.

**Figure 7:**
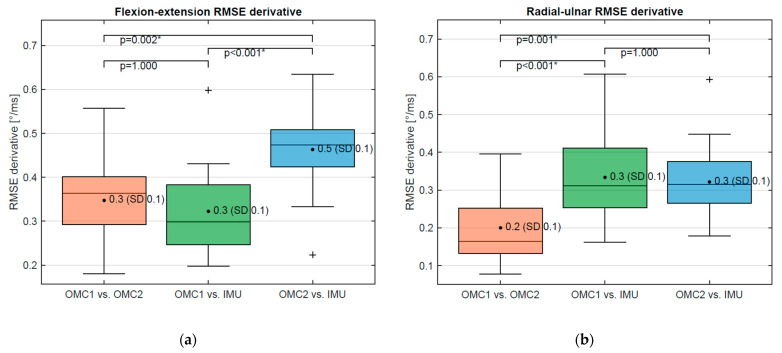
(**a**) RMSE of flexion–extension (filtered) derivative between OMC1, OMC2, and IMU acquisition systems and (**b**) RMSE of radial–ulnar deviation (filtered) derivative between OMC1, OMC2, and IMU acquisition systems. For Values marked as *, the difference is statistically significant. The symbols are statistical outliers and can be captioned as such.

For further comparison, the results are presented visually using boxplots of RMSE angles (Figure 6a,b) and derivatives (Figure 7a,b). For the flexion–extension angle, a significantly lower RMSE was found between OMC1 vs. IMU (8.3°, SD 2.5°) compared to OMC1 vs. OMC2 (11.7°, SD 3.9°) (*p* = 0.040) and compared to OMC2 vs. IMU (15.3°, SD 4.7°) (*p* = 0.00001). No significant difference was found for the RMSE of OMC1 vs. OMC2 compared to OMC2 vs. IMU. For the radial–ulnar deviation angle, the only statistical difference was found for the RMSE between OMC1 vs. OMC2 (7.1°, SD 4.1) and OMC1 vs. IMU (9.9°, SD 3.2) (*p* = 0.025).

For the flexion–extension derivative, no statistically significantly different RMSE was obtained when comparing OMC1 and OMC2 (0.35°, SD 0.10) and OMC1 vs. IMU (0.32°, SD 0.09) (*p*-value: 1.0) (Figure 7a). A statistically higher RMSE was found for the comparison of OMC2 vs. IMU (0.46°, SD 0.9) and OMC1 vs. OMC2 (*p* = 0.002) as well as OMC1 vs. IMU (*p* < 0.0001). For the radial–ulnar deviation derivative, the RMSE of OMC1 vs. OMC2 (0.20°, SD 0.09) is significantly smaller compared to RMSE OMC1 vs. IMU (0.33°, SD 0.11) (*p* = 0.0004) and RMSE OMC2 vs. IMU (*p* = 0.001) (Figure 7b). No statistically significant difference was observed between OMC1 vs. IMU and OMC2 vs. IMU (*p* = 1.0).

No statistically significant difference was found for the peak flexion–extension derivative (not filtered) for the OMC1 (12.6°/ms) and IMU (12.3°/ms) (*p* = 1.0). When measured with the OMC2, a significantly higher (*p* < 0.0002) maximum derivative (16.4°/ms) was found compared to the other two systems. For the average peak derivative in radial–ulnar deviation no significant difference was present between OMC1 (6.0°/ms) and IMU (6.8°/ms). The biggest value (*p* < 0.0013) was found for OMC2 (8.5°/ms).

**Figure 8:**
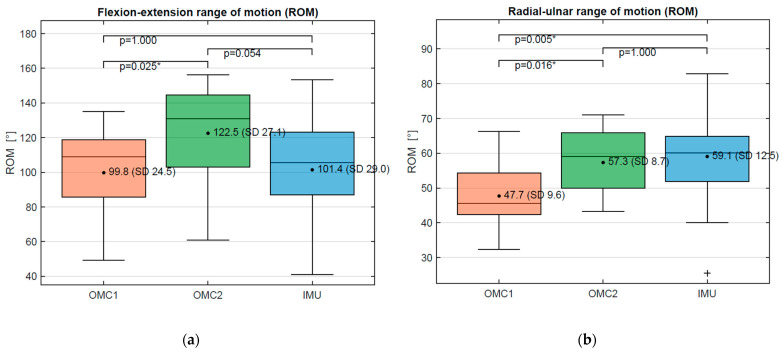
(**a**) Flexion–extension average ROM_DTM_ values for OMC1, OMC2, and IMU acquisition systems. (**b**) Radial–ulnar deviation average ROM_DTM_ values for OMC1, OMC2, and IMU acquisition systems. For Values marked as *, the difference is statistically significant.

There were large differences between the measured ROM_DTM_ of the different systems with coefficients of variation of 9–34%, as demonstrated in Table 1 and Figure 8. A systematic underestimation of the flexion ROM_DTM_ of 23° and 21°, respectively, was found for OMC1 and IMU compared to OMC2. The radial–ulnar ROM_DTM_ was 11° and 10° lower for the OMC1 compared to IMU and OMC2, respectively (Figure 8).

**Figure A2:**
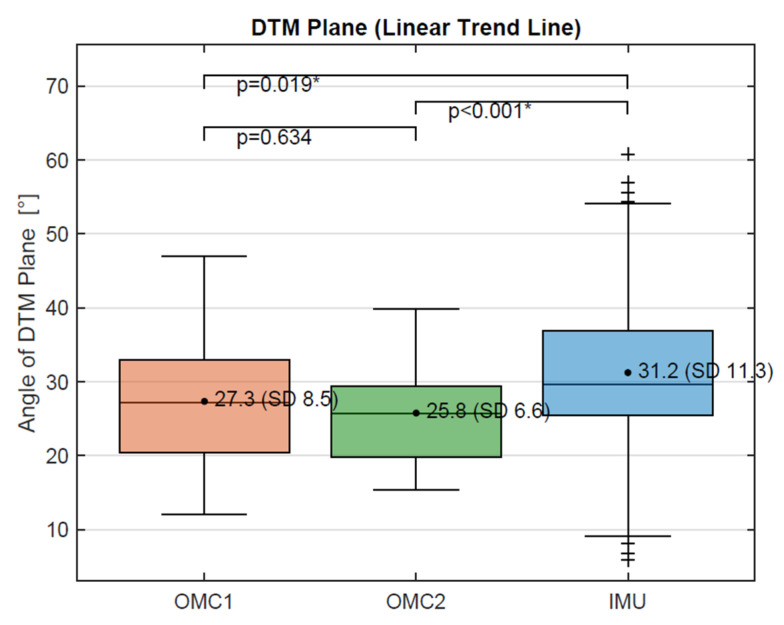
Angle of the calculated DTM plane by the three measurement methods OMC1, OMC2, and IMU. Statistically different values were found between IMU and OMC1 and between IMU and OMC2. For Values marked as *, the difference is statistically significant.

The calculated DTM plane, determined by the ratio of flexion–extension to radial–ulnar deviation angle (linear trend line) was 27.3° for the OMC1 and 25.8° for the OMC2 acquisition system, respectively. With 31.2°, the angle of the DTM plane for the IMU system differed significantly (*p* < 0.02) from the other two. A boxplot showing these results is presented in the Appendix A in Figure A2.

The duration of the throwing phases P2 and P_throw_ (during which the derivative is above 0.25°/ms) is significantly longer for the IMU compared to OMC1 (*p* < 0.0017) and OMC2 (*p* < 0.016) (Table 2). No significant differences in phase duration were found between OMC1 and OMC2 and between all measurement systems during P3 (Table 2).

Lower ROM_DTM_ flexion–extension values were found in this study than the ROM_BMT_ values found in our previous study of basic motion tasks [9]: the OMC1 measured only 73% of the ROM_BMT_ in this plane. For radial–ulnar deviation, OMC2 and IMU measured higher ROM_DTM_ than ROM_BMT_ values with 115% and 117% respectively. The ratios of the calculated ROM_DTM_ in this study compared to the ROM_BMT_ measurements in the two standard planes are shown for OMC1, OMC2, and IMU in Table 3.

**Figure 9:**
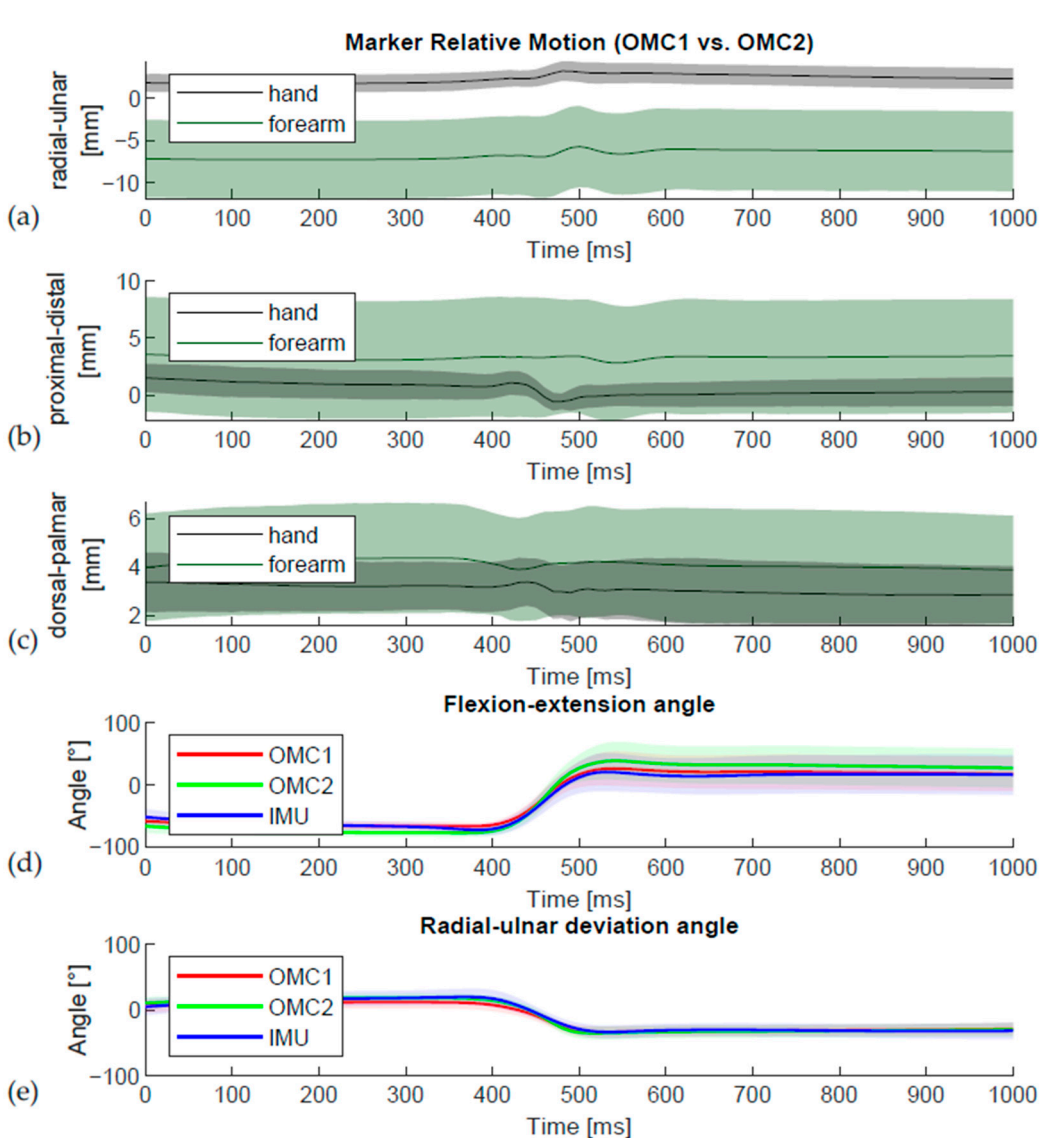
Visual representation of relative movement between the different OMC marker clusters against its reference position in (**a**) radial-ulnar, (**b**) proximal-distal and (**c**) dorsal-palmar direction. The wrist angles during the throw in (**d**) flexion-extension and (**e**) radial-ulnar deviation are displayed for the OMC1 markers in red, OMC2 markers in green, and IMU sensors in blue. A relative movement of the hand and forearm marker clusters of OMC1 and OMC2 was observed (**a**–**d**), which is associated with the throwing movement.

Relative movement between the skin marker clusters (OMC1) and the OMC markers directly on the IMU (OMC 2) was observed both on the hand and forearm (Figure 9). Bigger differences were observed in the forearm marker clusters. This is shown in more detail in Table 4.

## 4. Discussion

Since wrist mobility in the DTM plane plays an important role in the clinical evaluation of wrist function, a task-specific comparison between IMU and OMC was performed for the DTM with the intention of establishing a simple measuring tool for dynamic measurement of the active ROM in the DTM plane. The comparison was based on two different OMC marker sets, one with anatomical skin markers (OMC1), the other with markers attached directly to the sensor (OMC2). IMU values of all analyzed parameters are in the range of either OMC1 or OMC2. It therefore seems that the IMU is suitable to measure the DTM. As previously reported [9], the loss of marker visibility during trials represents a disadvantage of the OMC measurements. During one session, we encountered considerable problems with the OMC1 marker visibility during the DTM. The IMU enabled a continuous recording of the angle curves throughout all measurements. With regard to clinical use, the reliability of the IMU in acquisition is a major advantage.

However, compared to other tasks, generally rather low agreement between the systems was found for the DTM [9,20,21]. In comparison to the results published earlier on the basic motion tasks (BMT), where an average MAD of 6.5° is reported for the ROM in the anatomical planes [9], the average MAD was 15.9° for the ROM in the DTM between different systems. This corresponds to an average coefficient of variation of 7% for the BMT [9] and 21% for the DTM task, respectively. Only for the MAD between OMC1 and IMU in the radial–ulnar plane, a slightly smaller value (7.5°) was found for the DTM compared to the BMT (10.0°). All other differences between acquisition systems are considerably higher for the DTM task. Therefore, as described in previous studies [1,22], task-dependent deviations could also be confirmed with our specific setup.

Notably, the ROM_DTM_ acquired with the different marker sets also differs considerably. The found systematic difference in ROM_DTM_ of 25° in the flexion–extension plane and 11° in the radial–ulnar plane between OMC1 and OMC2 correspond to a variation of 22% and lie above the value that is considered clinically relevant [1]. In comparison, differences of less than 5% were reported for the BMT between the two marker sets [9]. The current results therefore highlight the importance of the marker location for motion analysis of the wrist. It is well known that the OMC measurement depends on the selected marker set, primarily due to skin movement artefacts [7,20,23] and that the pattern of the artefact is dependent on the task and joint angle, and movement speed, whereby the artefact increases with faster movement [23]. A previous study also found statistically significant differences in measured ROM between four different marker sets for the wrist [24], but only one of them deviated to a similar extent. Comparison with an imaging method would certainly be interesting to better understand the influence of marker or sensor locations and skin motion artefacts on the motion analysis of the wrist. A recent study by McHugh et al. found that the accuracy of optical motion capture in comparison with biplanar videoradiography is task dependent in the assessment of wrist movements; however, they did not investigate the DTM task [25]. The current results indicate that also task specific effects should be further analyzed.

This raises the question: What method, OMC1 or OMC2, do we choose as a basis for comparison with the IMU? When selecting the setup, we expected the calculated joint angles derived from the markers on the sensors (OMC2) would have better agreement with the IMU, as this method actually tracks the movements of the sensors. The disadvantage of constrained methods, i.e., where the markers are fixed to an object, is that systematic artefacts in respect to the underlying joint can be introduced by inertial effects [23]. Given the rather large weight of the sensor, such inertial effects must be taken into account when evaluating OMC2. On the other hand, skin displacement artefacts of the markers relative to the sensors are to be expected for OMC1. However, OMC1 takes into account the principle of free moving skin markers and the largest possible cluster radius, which are recommended to reduce the effect of skin movement artifacts [7,23]. Therefore, from a biomechanical point of view, marker placement OMC1 seems more ideal for estimating wrist angles whereas OMC2 offers more direct measurement of the sensor movements.

The proportion between flexion–extension and radial–ulnar deviation serves as a measure for the orientation of the DTM plane. It is similar when determined for OMC1 (27.4°) and OMC2 (26.0°) but differs when measured with the IMU (33.5°). The OMC results are very similar to the angle determined by Kane et al. for the DTM plane on intact wrist specimens with 26.6° [26]. The difference between OMC and IMU could be an indication of deviations in the anatomical planes defined by the different acquisition systems. In the evaluation of the IMU, the anatomical planes are determined by the placement of the sensors, in contrast to the functional calibration for both OMC methods. The functional determination of the joint coordinate system has proven to be more accurate and less sensitive to inter-observer differences in previous studies [27,28]. Hence, whether an optimized alignment of the sensor coordinate systems to the anatomical planes, either by a more sophisticated physical alignment of the sensors on the segments or by a virtual alignment using a functional calibration, would increase the agreement between joint angles of OMC and IMU remains to be investigated in future studies.

A significantly “longer duration of the throwing-phase” P_throw_ (defined as the time when the derivative of the angle curve is above a certain threshold for more than 30 ms) could be shown in the IMU measurements when compared to OMC1 and OMC2. This difference is mainly caused by a significantly longer duration of P2, the phase from the beginning of the throwing phase (T1) to the maximum derivative (T2). No significant differences in the duration were found for P3, the phase from the maximum derivative (T2) to the end of the throw (T3), or between the two OMC methods. Since all systems recorded the same trial simultaneously, this difference in duration of the “unstable” phase of the signal is unexpected and must have been caused by effects of the different measurement methods. In the following, we have examined the wrist angle curves for the different measurement systems in more detail: in the qualitative observation, the IMU signal shows an initial wrist movement towards radial-extension followed by a movement in the direction of ulnar-flexion at the beginning of the throwing movement (T1). In most cases, the OMC measurements indicate that the test persons remain in a radial-extended wrist position for a short time before performing the throw in the direction of ulnar-flexion. The observed “backswing movement” of the IMU angles is in accordance with the longer duration of the accelerated phase of the IMU signal. In the OMC videos showing the marker positions, no backswing movement could be detected visually, but it was observed that in some cases the throw was started by a movement in the elbow. It should be further investigated whether the IMU measurement is affected by such additional movements of adjacent joints that possibly cause an acceleration of the forearm and palm segments/ sensors without relative movement in the wrist. One hypothesis is that the IMU fusion algorithm relies more on the acceleration than on gyroscopic motion. Hence, the linear backward motion before throwing the dart is understood as an orientation change by the IMU. However, this assumption needs further studies to be clarified. Nevertheless, it is still unclear why the OMC wrist angles remain unchanged whereas the IMU signals have an additional “ripple” prior to the ulnar-flexion slope and which pattern corresponds to the actual movement of the test persons.

The execution of movements was very different between the individual test subjects in terms of timing (SD P_throw_ 66–142 ms) and displacement (SD ROM_DTM_ flexion–extension 24°–28°). Although they were instructed to perform the throw by a maximal movement of the wrist, some individuals did not reach flexion at all, but remained in an extended wrist position during the whole throwing movement. As all test subjects are healthy volunteers, it seems not all of them have exploited their maximal wrist mobility during the DTM. Hence, the maximum ROM_DTM_ values should clinically be interpreted with caution. A slower execution of a simulated movement in the DTM plane should be considered to make it easier for the test persons to use the full range of wrist motion and improve the measurement accuracy of the systems. The small amount of test subjects that performed a task with very high variability poses a limitation for the comparison of the systems and the interpretation of individual cases. Nevertheless, the large inter-subject tasks variability is a viable scenario for a clinical application, as patients are expected to have even larger differences in movement performance. In order to obtain more specific values for the measurement accuracy with regard to varying wrist mobility, it should be further investigated in a larger number of subjects whether the agreement of the systems differs depending on the motion execution of the DTM, e.g., in subjects with small ROM.

The post hoc temporal alignment and cutting of the signals is a limitation of the study. For trials with big differences in measured values between the systems, a subsequent alignment of the angular curves is difficult. Despite being carefully selected, the used alignment method might have influenced the results. Furthermore, the RMSE depends on selection of the duration of the throwing phase, whereas the ROM is less sensitive in this respect. In selecting the point of interest, we have tried to meet the following criteria: maximum derivative is included, minima and maxima of the signals are included, all trials are cut to the same duration, the movement from the starting position on the table to the initial position of the throw and after the throw going back to the table should not be evaluated. Due to the very different movement executions of the test persons, a compromise had to be made between the different criteria. To ensure a focus on the relevant movement in all trials, the maximum angle was cut at the end of a few trials. In the end, it remains a subjective decision which part of the signal is considered to be useful for the evaluation.

For the DTM task measured with OMC1, subjects used 72% of their maximum flexion–extension ROM_DTM_ and 91% of their maximum radial–ulnar deviation ROM_DTM_ (Table 3). These values are very similar to the results of the 20 healthy subjects in a previous study (68% und 86% respectively) [2]. Remarkably, the radial–ulnar deviation ROM_DTM_ during the DTM is higher than the ROM in the anatomical radial–ulnar plane [9] for the OMC2 (116% of BMT) and IMU (122%) acquisition but not for OMC1 (91% of ROM_BMT_). These unexpected values could possibly be caused by displacement of the skin due to inertial effects, which are a recognized problem for rigid methods of marker attachment [23] caused by the weight of the sensor during the fast movement of the DTM. In accordance, the observed relative movement between the OMC1 and OMC2 marker clusters depicted in Figure 9 also indicates skin movement artefacts.

Without imaging techniques, the contribution of the individual components to the overall error cannot be differentiated. As the differences compared to movements in the anatomical planes are bigger [9], it could be shown that the differences are task dependent. This is in agreement with previous observations on the comparison between IMU und OMC [21,29] as well as task dependent patterns of error associated with soft tissue artefacts [23]. The DTM presented here is a greater challenge for the systems than movements in the pure anatomical planes [9]. The DTM is faster and consists of a combined movement that is performed in an oblique wrist plane. The deviations in the DTM plane are an indication of different orientations of the joint coordinate systems between OMC and IMU. The alignment of the joint coordinate system is an important factor in reducing kinematic cross-talk [27], which may have a greater effect when moving in an oblique plane.

## 5. Conclusions

This study clearly demonstrates the importance of the marker positioning for the OMC measurement. It also shows the difficulties in choosing the basis of comparison for the validation of the IMU as well as the limited comparability of the kinematic results derived from different wrist OMC marker sets or different measurement systems. For the OMC system, the measured ROM with the sensor markers is systematically larger than with the skin markers, but the orientation of the DTM plane is similar. The DTM plane determined with the IMU deviates from OMC1 and OMC2. The alignment of the sensors therefore needs further investigation. Without an imaging technique, however, it is neither possible to quantify the effect of possible sources of error nor to distinguish which resulting motion pattern acquired by the different measurement methods corresponds best to the actual wrist movement of the test persons.

The DTM is considered to be the primary plane of wrist movement in daily activities; therefore, DTM measurements might be an important indicator for assessing wrist function. With the IMU, it is possible to measure dynamic and coupled wrist movements, such as the DTM, and the observed results are promising for future clinical applications. Hence, the IMU provides a tool for the evaluation of DTM ROM that can be easily implemented in clinical evaluation of this highly relevant wrist movement.

## Figures and Tables

**Table 1:** Summary of the mean (± SD) ROM_DTM_ in both the flexion–extension and radial–ulnar deviation planes for each system, as well as the mean absolute difference (MAD) and the coefficient of variation (CV) between the different systems.

	ROM OMC1	ROM OMC2	ROM IMU	MAD OMC1 vs. OMC2	MAD OMC1 vs. IMU	MAD OMC2 vs. IMU	CV OMC1 vs. OMC2	CV OMC1 vs. IMU	CV OMC2 vs. IMU
Mean and SD Flexion–Extension	99.8° (SD 24.5°)	122.5° (SD 27.1°)	101.4° (SD 29.0°)	22.8° (SD 7.9°)	8.2° (SD 4.0°)	23.0° (SD 8.8°)	21.1% (SD 7.5%)	8.8% (SD 5.0%)	22.4% (SD 12.3%)
Mean and SD Radial–ulnar deviation	47.7° (SD 9.6°)	57.3° (SD 8.7°)	59.1° (SD 12.5°)	10.6° (SD 7.3°)	17.3° (SD 8.0°)	11.1° (SD 5.1°)	20.9% (SD 14.6%)	33.6% (SD 17.3%)	20.2% (SD 12.0%)

**Table 2:** Mean values of the different throw phases depicted as P2 and P3 and the total throw time (P_throw_) and standard deviations (SD) for all three measurement systems.

Measurement System	P2	P3	P_throw_
OMC1	113 ms (SD: 20 ms)	183 ms (SD: 73 ms)	295 ms (SD: 77 ms)
OMC2	115 ms (SD: 18 ms)	187 ms (SD: 65 ms)	301 ms (SD: 67 ms)
IMU	162 ms (SD: 9 3ms) *^/^**	192 ms (SD: 93 ms)	354 ms (SD: 138 ms) *^/^**

* Significant difference to OMC1; ** Significant difference to OMC2.

**Table 3:** Proportion between the maximum ROM_BMT_ in the anatomical planes (flexion–extension and radial–ulnar deviation) and the ROM during the DTM.

ROM_DTM_ vs. ROM_BMT_	OMC1	OMC2	IMU
Flexion–Extension DTM vs. BMT	73%	87%	81%
Radial–Ulnar DTM vs. BMT	93%	115%	117%

**Table 4:** Mean absolute difference (MAD) and peak change in centroid distance (peakD) between Table 1 and OMC2 marker clusters during different tasks: dart throwing motion (DTM), flexion–extension (FE), and radial–ulnar deviation (RU).

Segment (OMC1 vs. OMC2 Marker Cluster)	DTM		FE		RU	
	MAD	peakD	MAD	peakD	MAD	peakD
Hand	2.7 *^/^**	5.3 *^/^**	1.3	2.8	1.4	2.3
Forearm	3.0	4.7 *	2.4	3.4	2.4	3.2

* Significant difference to RU task; ** Significant difference to FE task.

**Figure A3:**
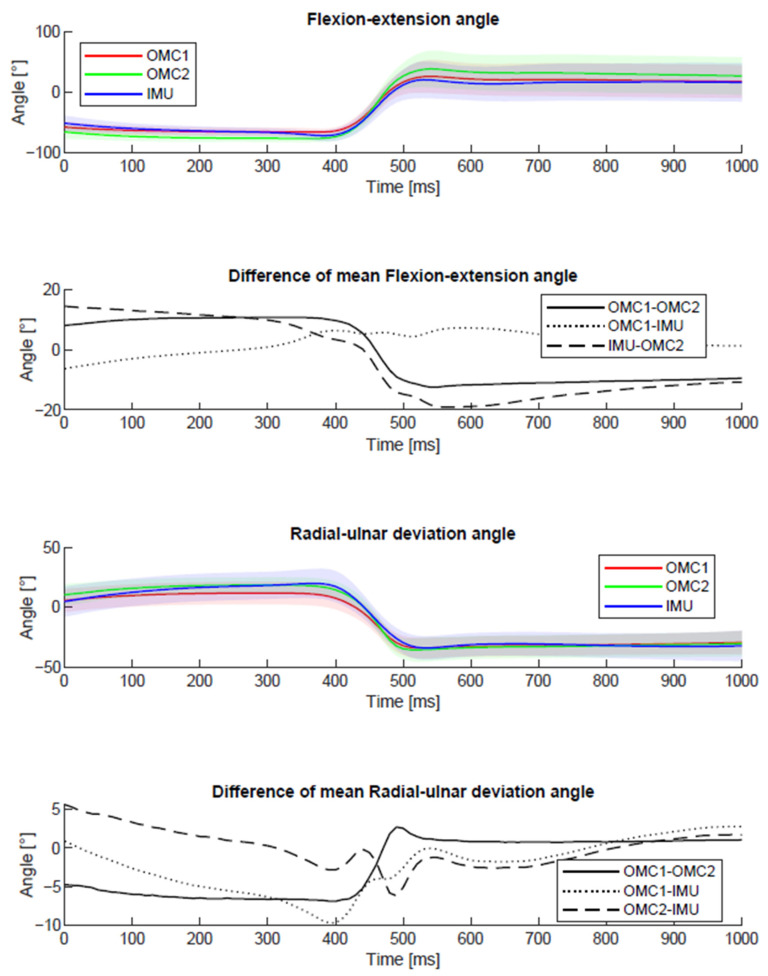
Visual representation of the effective angle curves and differences between the measurement methods during the trials.

## Appendix A

Figure A1 shows in detail the comparison between the different measurement methods for each individual test person and separated for both measurement days. A visual representation of the calculated plane in which the DTM was performed, determined by the ratio of flexion–extension to radial–ulnar deviation angle, is given in Figure A2. Figure A3 shows the effective angle curves obtained by the different measurement methods OMC1, OMC2, and IMU as well as the differences between them in the course of the trials in temporal alignment.
